# Supplementary material for: The interactome of intact mitochondria by cross-linking mass spectrometry provides evidence for coexisting respiratory supercomplexes
Source: Mol Cell Proteomics. 2017 Dec 8;17(2):216–32. doi: 10.1074/mcp.RA117.000470 (PMC5795388; doi:10.1074/mcp.RA117.000470)
Supplement: Supplemental Data [file supp_RA117.000470_133922_0_supp_23978_fzffwf.zip › spectra_annotation/mito_spectra_annotation/264-1-4-1-4-1.pdf]

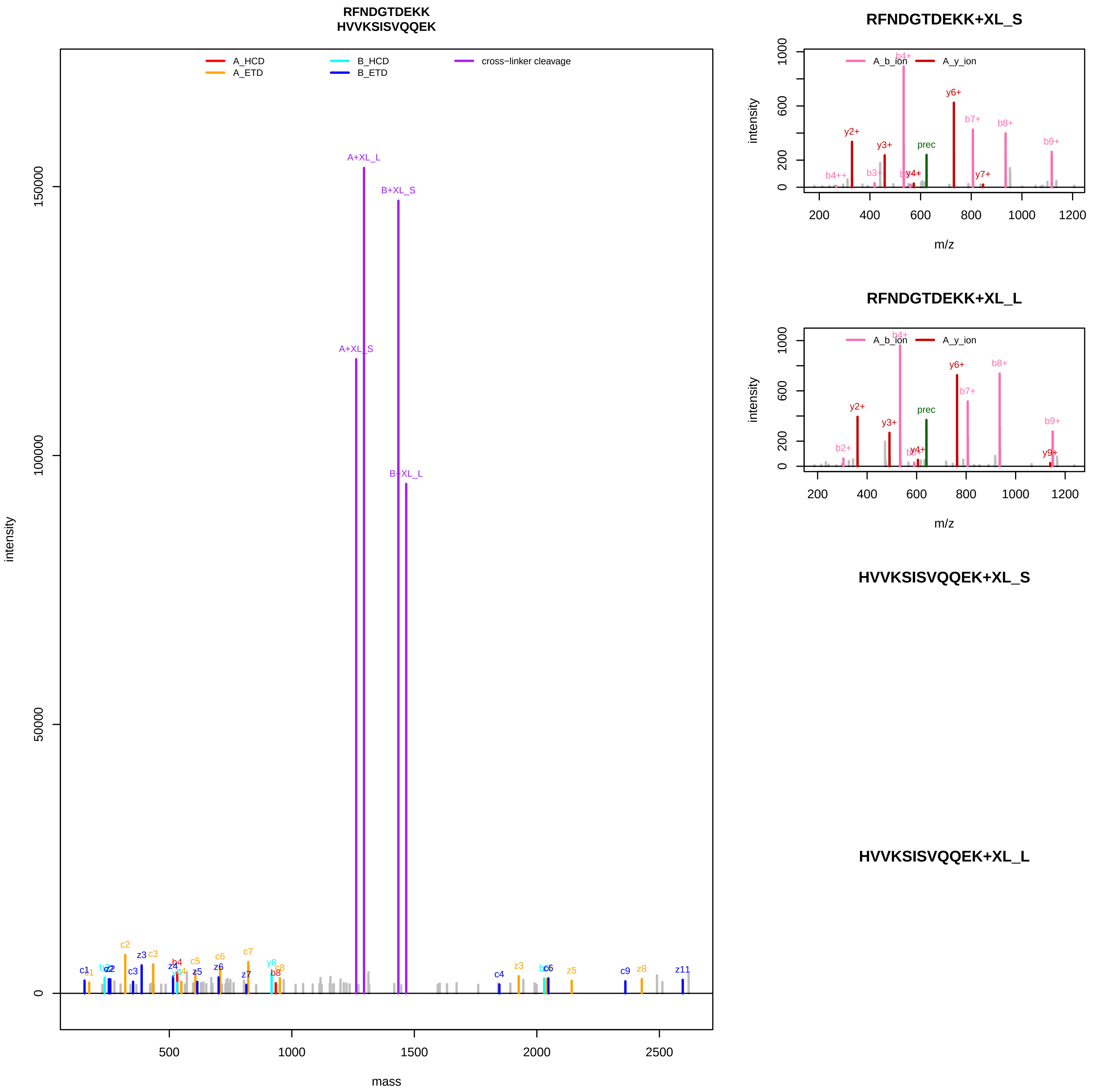
RFNDGTDEKK
HVVKSISVQQEK
A_HCD
A_ETD
B_HCD
B_ETD
cross−linker cleavage
A+XL_L
B+XL_S
A+XL_S
B+XL_L
intensity
mass
0
50000
100000
150000
500
1000
1500
2000
2500
c1
c2
c3
z3
z4
b4
c5
z5
c6
z6
c7
z7
y8
b8
c8
c4
z3
c6
z5
c9
z8
z11
RFNDGTDEKK+XL_S
A_b_ion
A_y_ion
b4+
y6+
y2+
y3+
prec
b7+
b8+
b9+
b4++
b3+
y4+
y7+
m/z
RFNDGTDEKK+XL_L
b2+
y9+
HVVKSISVQQEK+XL_S
HVVKSISVQQEK+XL_L
